# *PLoS Genetics* Turns Three: Looking Back, Looking Ahead

**DOI:** 10.1371/journal.pgen.1000135

**Published:** 2008-07-25

**Authors:** Wayne N. Frankel, Gregory S. Barsh

**Affiliations:** 1 The Jackson Laboratory, Bar Harbor, Maine, United States of America; 2 Departments of Genetics and Pediatrics, Stanford University School of Medicine, Stanford, California, United States of America

*PLoS Genetics* is three years old this month—a milestone worth celebrating! As we do, and as we recognize all who have helped us reach this point in time, we thought this would be a good opportunity to share with you a summary of our brief history and a look ahead.

Our original intent was to provide an open-access journal for the community that would “reflect the full breadth and interdisciplinary nature of genetics and genomics research by publishing outstanding original contributions in all areas of biology.” Now, three years later, all of us on the Editorial Board are very pleased with the breadth of topics covered and with the diversity of approaches, organisms, and systems. Going forward, *PLoS Genetics* will continue to be a journal by and for the entire genetics and genomics community.

Together with diversity, an essential component of the journal is our emphasis on work that is rigorous, that significantly advances the field, and that has broad scientific appeal. These assessments (particularly the latter two) are not always straightforward, especially when advances in technology, knowledge, and/or public interest stimulate a large number of manuscript submissions from a particular area. In the past year, for example, the journal has naturally seen an increase in the number of submissions that deal with the cataloging and analysis of large-scale sequence, expression, and phenotype data, including meta-analyses and development of new computational methods. As has been the case to date, *PLoS Genetics* will actively consider such studies, while encouraging work that also contains some empirical validation or exploration, for example with “wet bench” experimentation or application to a real dataset. This criterion is not intended as a pejorative judgment, but rather echoes the consensus of the Editorial Board that published papers should continue to bring significant new biological insight to a respective field. More importantly, this example reflects the nature of scientific achievement—as a community, we should anticipate changes in the types of experiments we do and the types of papers we publish, with an overall guiding principle to learn more about how genes and genome sequences influence the world around us. Accordingly, we expect to regularly evaluate the journal's scope from a practical perspective and to then clearly guide prospective authors in terms of specific types of manuscripts we encourage and discourage.

Judged by the rising rate of submissions, the journal has been successful (Figure 1). Certainly, the transition to publishing only online, since January 2006, had no negative effect on submissions or downloads, supporting the contention that most scientists use online sources for their research when there is a choice. We are also pleased that the average time to rendering the initial decision for peer-reviewed research articles is just over 30 days—quite decent for a journal edited entirely by working scientists. This is a credit both to the diligence of our Associate Editors and to the good will of external peer reviewers. Finally, as our Editorial Board has matured and the journal finds its place in the community, the acceptance rate of full manuscript submissions has settled in at around 25%. Considering that this rate includes many manuscripts that were greatly improved as a result of very thoughtful peer review and author response, we think that this is a suitable outcome for a journal that endeavors to balance scientific quality and novelty with fairness.

**Figure 1:**
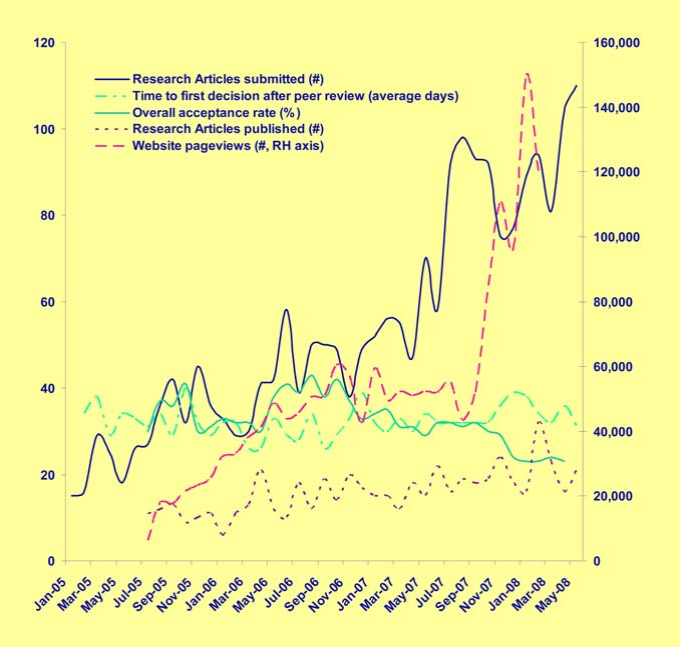
*PLoS Genetics* Manuscript Summary Statistics.

One price of success is, of course, an increased workload, which has led to the expansion of the Editorial Board at all levels. Over three years, *PLoS Genetics* has steadily added new Associate Editors to the Board, from 29 (in July 2005) to 59 at the time of writing. The current structure—with distinct groups of senior editors for the fields of epigenetics, evolution, natural variation, and gene expression, and a group for the remainder of submissions—continues to work well, with substantial communication among all of us to ensure balance and consistency. Several new senior editors, who (along with us) make “triage-level” decisions, who oversee Associate Editor assignments, and who review editorial decisions, have joined the Board (or soon will), and this will be a critical next step in ensuring the journal's ability to consider the work the community entrusts to us. As this Board expansion begins, we would like to thank our charter senior editors who worked hard over the past year to make *PLoS Genetics* a strong journal that retains the community spirit: Section Editors Greg Gibson (natural variation and gene expression), Gil McVean (evolution), and Wolf Reik (epigenetics).

The inclusion of Reviews, Perspectives, and Interviews, collectively known as “front matter” in the industry, contributes in many ways to the impact of a journal—both with respect to the content provided as well as, more literally, the part such articles play in the calculation of the journal's Impact Factor. While great value is often placed on the latter, the Editors of *PLoS Genetics* also see the value of our front matter articles in their educational content (for example, to aid newcomers to a field) and in their ability to foster community discussion. Indeed, open-access publishing greatly facilitates both goals. *PLoS Genetics* has had a committed team of front-matter editors, including Lizzy Fisher, Nico Katsanis, Marcy MacDonald, and Susan Rosenberg (who commission and edit Review Articles), as well as Jane Gitschier, who single-handedly invites, writes, and edits our unique series of Interviews. As the journal matures and we fine-tune the “front matter” section, readers can expect to see a diversity of recruited articles appearing in the coming year. Hopefully, our readers will take full advantage of our recent migration to the Topaz publishing platform and use the Web tools now available to participate in discussions on and about our published content.

One of the attributes of the Editorial Board, including all senior editors, is that we are working scientists who volunteer their “free” time (as if there is such a thing…) for the cause. In other words, manuscripts are not only reviewed externally by peers, but editorial decisions are also made by individuals in the trenches. Our impression is that, whether manuscripts are published or eventually turned away, authors generally take comfort in this type of editorial process. That is, our Board members know well the thrill of victory and the agony of defeat in their own efforts. Although some authors may ultimately be disappointed by our decisions, we cannot imagine a more equitable system of scientific review.

Most of the work on the ground is done by the Associate Editors of Research Articles, an extremely dedicated and thoughtful group, and the peer reviewers. We are grateful to all of our Associate Editors, and particularly would like to acknowledge those who have stuck with *PLoS Genetics* from the beginning, namely Gonçalo Abecasis, David Allison, David Beier, Andy Clark, Susan Dutcher, Jonathan Flint, Claire Fraser-Liggett, Greg Gibson, Takashi Gojobori, Jim Haber, Scott Hawley, Yoshihide Hayashizaki, Stuart Kim, Leonid Kruglyak, Trudy Mackay, Susan Mango, Mary Mullins, Harry Orr, Molly Przeworski, Wolf Reik, Derry Roopenian, Mike Snyder, David Stern, Barbara Trask, David Valle, and Veronica van Heyningen. On behalf of our Editorial Board, we would also like to thank over 2,450 reviewers and 60 or more guest Associate Editors who have very generously donated their time over the past 3 years. Their names can be found together in Table S1.

Finally, on behalf of all the editors at *PLoS Genetics*, we thank you for your support of the journal—and we look forward to serving you in the future.

## Supporting Information

Table S1 Reviewers and Guest Associate Editors, January 2005–May 2008
(0.08 MB PDF)

