# Supplementary material for: PLoS Genetics Turns Three: Looking Back, Looking Ahead
Source: PLoS Genet. 2008 Jul 25;4(7):e1000135. doi: 10.1371/journal.pgen.1000135 (PMC2444048; doi:10.1371/journal.pgen.1000135)
Supplement: Table S1 — Reviewers and Guest Associate Editors, January 2005–May 2008 (0.08 MB PDF) [file pgen.1000135.s001.pdf]

Table S1. Reviewers and Guest Associate Editors, January 2005–May 2008.

We thank the following people who have very generously donated their time over the past three years in the capacity of reviewers and/or guest Associate Editors for *PLoS Genetics*:

Abdel-Malek, Zalfa	Andersen, Gitte	Attie, Alan
Abecasis, Gonçalo	Anderson, Clark	Aubin, Jane
Abel, Laurent	Anderson, Erik	Auger, Don
Acharya, Jairaj	Anderson, James	Auwerx, Johan
Achtman, Mark	Anderson, Lorinda	Avery, Leon
Ackermann, Martin	Andersson, Bjorn	Avner, Philip
Adams, David	Andersson, Leif	Avraham, Karen
Adams, Keith	Andersson, Siv	Awadalla, Philip
Adams, Mark	Andino, Raul	Axel, Richard
Adelson, Dave	Andolfatto, Peter	Aylor, David
Adler, Adam	Andrews, Brenda	Azuara, Veronique
Agaisse, Hervé	Andrews, Justen	Babitzke, Paul
Agami, Reuven	Angela, Tyner	Bachtrog, Doris
Aguilera, Andres	Anholt, Robert	Bader, Joel
Ahmad, Kami	Antebi, Adam	Badge, Richard
Ahmed, Shawn	Antonovics, Janis	Badger, Jonathan
Ahn, Ji-Hoon	Aparicio, Oscar	Baehrecke, Eric
Ahringer, Julie	Aparicio, Samuel	Bähler, Jürg
Aiba, Hiroji	Aragon, Luis	Bahlo, Melanie
Aitman, Timothy	Arbeitman, Michelle	Bahn, Sabine
Akashi, Hiroshi	Archer, Simon	Baines, John
Akey, Joshua	Argyropoulos, George	Baker, Bruce
Akhurst, Rosemary	Arking, Dan	Baker, Scott E.
Albani, Salvatore	Armanios, Mary	Balding, David
Albert, Reka	Armbrust, E. Virginia	Balling, Rudi
Albertson, Donna	Arnett, Donna	Balloux, Francois
Alcais, Alexandre	Arnold, Arthur	Bally-Cuif, Laure
Allayee, Hooman	Arnosti, David	Balmain, Allan
Allison, David	Artandi, Steven	Banting, George
Allsopp, Basil	Artavanis-Tsakonas, Spyros	Barbash, Daniel
Almouzni, Genevieve	Asai, David	Barbour, Alan
Al-Sady, Bassem	Ashrafi, Kaveh	Barcellos, Lisa
Altman, Russ	Ashworth, Alan	Barciszewski, Jan
Altshuler, David	Ason, Brandon	Bardeesy, Nabeel
Amacher, Sharon	Ast, Gil	Bargmann, Cori
Amasino, Rick	Aster, Jon	Bargonetti, Jill
Amatruda, James	Atchison, Michael	Barlow, Denise
Amiel, Jeanne	Atkinson, John	Barnholtz-Sloan, Jill
Amos, Christopher	Atomi, Haruyuki	Barr, Maureen

Barral, Yves	Bejsovec, Amy	Birchler, James
Barre, François-Xavier	Bell, Douglas	Bird, Adrian
Barres, Ben	Bell, Graeme	Birnbaum, Kenneth
Barrick, Jeffrey	Bell, Steve	Birney, Ewan
Barroso, Ines	Bellefroid, Eric	Bishop, Douglas
Barsh, Greg	Bellen, Hugo J.	Bjornsti, Mary-Ann
Bartel, David	Bell-Pedersen, Deborah	Blackshaw, Seth
Bartke, Andrzej	Belmont, John	Blackwell, T. Keith
Bartlett, Chris	Belshaw, Robert	Blake, Judith
Bartolomei, Marisa	Benavente, Ricardo	Blanchette, Mathieu
Barton, Anne	Bender, Judith	Blandin, Stephanie
Barton, Michelle	Bender, Michael	Blaser, Martin
Barton, Nick	Benfey, Philip	Blelloch, Robert
Barzilai, Nir	Benjamin, Emelia	Bloom, Kerry
Basler, Konrad	Berger, Frederic	Blower, Michael
Bass, Brenda	Bergman, Casey	Blumenstiel, Justin
Bastian, Boris	Bergmann, Dominique	Blumenthal, Thomas
Bataillon, Thomas	Bergstrom, Carl	Bock, August
Batalov, Serge	Bernander, Rolf	Bock, Christoph
Bateman, John	Bernardi, Giorgio	Boehm, Thomas
Battelle, Barbara	Bernards, Rene	Boehnke, Michael
Battistuzzi, Fabia	Bernstein, Brad	Boeke, Jef
Batzer, Mark	Berriman, Matthew	Boerner, Valentin
Baugh, Ryan	Bertone, Paul	Boffelli, Dario
Baum, Christopher	Bertorelle, Giorgio	Bohnert, Hans
Baumann, Peter	Bertuch, Alison	Bolland, Silvia
Baumeister, Ralf	Besansky, Nora	Bonini, Nancy
Baylin, Stephen	Bestor, Timothy	Bonomo, Robert
Beal, Flint	Betancourt, Andrea	Boone, Charles
Beato, Miguel	Betel, Doron	Borde, Valerie
Beaty, Terri	Betran, Esther	Bordenstein, Seth
Beaudet, Arthur	Beutler, Bruce	Borevitz, Justin
Beaumont, Mark	Bhattacharya, Shoumo	Borts, Rhona
Beck, Stephan	Bichet, Daniel	Bosco, Giovanni
Becker, Kevin	Bickel, Sharon	Bosenberg, Marcus
Becker, Peter	Bickmore, Wendy	Boss, Jeremy
Beckstead, Robert	Bidichandani, Sanjay	Botas, Juan
Beckwith, Jon	Biek, Roman	Botstein, David
Bedell, Mary	Bieker, James	Boulton, Simon
Beemon, Karen	Bielinsky, Anja-Katrin	Bour'chis, Deborah
Beer, Michael	Bier, Ethan	Bowcock, Anne
Beer, Mike	Biessmann, Harald	Bowman, John
Begun, David	Biggins, Sue	Boyko, Adam
Beier, David	Bilder, David	Bradley, Dan
Bejerano, Gill	Birch, Paul	Brash, Alan

Braun, Robert	Burgess, Sean	Casares, Fernando
Bray, Dennis	Burgoyne, Paul	Casella, George
Breen, Matthew	Burke, Dan	Cashmore, Tony
Brehm, Alexander	Burkholder, William	Castle, John
Brem, Rachel	Burmeister, Margit	Catcheside, David
Brendel, Volker	Burt, Austin	Caulfield, Mark
Brennan, Paul	Burtis, Kenneth	Cavalli, Giacomo
Brenner, Charles	Bushel, Pierre	Cedar, Howard
Bresnick, Emery	Bushman, Frederic	Ceman, Stephanie
Bridges, Louis	Bussemaker, Harmen	Chaconas, George
Britt, Steven	Bussey, Howard	Chadwick, Brian
Brivanlou, Ali	Bustamante, Carlos	Chaillet, J Richard
Brock, Hugh	Butlin, Roger	Chaix, Raphaelle
Brockdorff, Neil	Byers, Peter	Chalker, Doug
Brodkin, Ted	Cagan, Ross	Chan, David
Brodsky, Michael	Caicedo, Ana	Chan, Simon
Broman, Karl	Calabrese, Peter	Chan, Yvonne
Brookfield, John	Caldecott, Keith	Chang, Howard
Brosius, Jürgen	Calin, George	Chang, Hwan-You
Brown, Grant	Calos, Michelle	Chang, Sandy
Brown, Matthew	Calvi, Brian	Chanock, Stephen
Brown, Myles	Camerini-Otero, Dan	Chapman, Tracey
Brown, Powel	Camilloni, Giorgio	Charlesworth, Brian
Brown, Steve	Camp, Nicola	Charlesworth, Deborah
Brown, Susan	Campbell, Malcolm	Charlier, Carole
Brown-Kennerly, Victoria	Campbell, Shelagh	Chartrand, Pascal
Brudno, Michael	Camper, Sally	Chattoraj, Dhruba
Brueckner, Katja	Canitrot, Yvan	Chaudhury, Abed
Bruemmer, Dennis	Cantor, Rita	Chen, John
Brunet, Anne	Capecchi, Mario	Chen, Liang
Brunner, Han	Capy, Pierre	Chen, Meng
Brusic, Vladimir	Carey, Vincent	Chen, Rey-Huei
Bryan, Jenny	Carlborg, Orjan	Chen, Z. Jeffrey
Brzeski, Jan	Carlson, Christopher	Cheng, Hans
Bubulya, Tom	Carmena, Mar	Cheng, Keith
Bucan, Maja	Carmo-Fonseca, Maria	Chesler, Elissa J
Buchner, Erich	Carninci, Piero	Chess, Andrew
Buchrieser, Carmen	Carpenter, Adelaide	Cheung, Vivian
Buck, Michael	Carr, Antony	Chiang, Chin
Buckler, Edward	Carrington, Mary	Chiaromonte, Francesca
Burch, Christina	Carroll, Raymond	Chien, Chi-Bin
Burchard, Esteban	Carroll, Sean	Chikhi, Lounes
Burdine, Rebecca	Carter, Dee	Chinnery, Patrick
Burge, Chris	Cartwright, Paulyn	Chisholm, Andrew
Burgess, Daniel	Casadesus, Josep	Chistiakov, Dimitry

Chitnis, Ajay	Constantini, Frank	Csuros, Miklos
Cho, Judy	Contreras-Moreira, Bruno	Cullen, Bryan
Cho, Ken	Cookson, Bill	Cummins, Theodore
Choi, Kyunghee	Cookson, Mark	Cunninghame Graham, Deborah
Christensen, Kaare	Coombes, Kevin	Cuppen, Edwin
Christians, Julian	Coonrod, Scott	Curcio, M. Joan
Christiansen, Jeff	Coop, Graham	Daha, Mohammed
Christophides, George	Cooper, Alan	Dahary, Dvir
Chui, David	Cooper, Julie	Daly, Mark
Church, Deanna	Cooper, Max	Daly, Michael
Church, George	Cooper, Thomas	Dang, Chi
Churchill, Gary	Copeland, Neal	Darnell, James
Cichutek, K	Copeland, Paul	Darvasi, Ariel
Claas, Frans	Copeland, William	Datta, Milton
Clark, Amander	Copenhaver, Gregory	Daubin, Vincent
Clark, Andrew	Cordaux, Richard	Davidson, Nicholas
Clark, Clifford	Cordell, Heather	Davies, Jamie
Clark, Peter	Corey, David	Davies, Kay
Clarke, Cathy	Cormier-Daire, Valerie	Davis, Richard
Clarke, Robert	Cornejo, Omar	Davison, Daniel
Clegg, Michael	Cornet, François	Davisson, Muriel
Cleveland, John	Cornforth, Michael	Davuluri, Ramana
Cloninger, Robert	Cossins, Andrew	Dawe, Kelly
Cobrinik, David	Costa, Jose	Dawes, Ian
Coffin, John	Costello, Joseph	Dawson, Deborah
Coghlan, Avril	Coté, Jacques	De Andrade, Mariza
Cohen, Barak	Cotsapas, Chris	De Bakker, Paul
Cohen, Paula	Cotton, Richard	De Boer, Rob
Cohen, Stephen	Counter, Christopher	De Celis, Jose F.
Cohen-Fix, Orna	Covacci, Antonello	De Haan, Gerald
Cohn, Martin	Cover, Timothy	De Jager, Philip
Colditz, Graham	Covert, Sarah	De Koning, D
Cole, Douglas	Cowell, John	De Krijger, Ronald
Coller, Hilary	Cox, David	De la Chapelle, Albert
Collins, Kathleen	Cox, Michael	De Laat, Wouter
Colot, Vincent	Cox, Nancy	De Lozanne, Arturo
Comai, Luca	Crabtree, Gerald	De Massy, Bernard
Comuzzie, Anthony	Crawford, Dana	De The, Hugues
Conant, Gavin	Crawford, Douglas	De Veylder, Lieven
Condie, Brian	Crease, Teri	De Visser, J. Arjan G. M.
Cone, Karen	Crespi, Martin	Dean, Ann
Conlan, Sean	Criscuolo, Francois	Dean, Caroline
Conley, Mary Ellen	Cross, Fred	Deen, Peter
Conrad, Don	Cross, Jay	DeGregori, James
Conradt, Barbara	Csermely, Peter	Deininger, Prescott

Dekker, Job	Drayna, Dennis	Emili, Andrew
Delmotte, François	Dresser, Michael	Eng, Charis
Delseny, Michel	Driscoll, Monica	Engel, James
Delsuc, Frederic	Dröge-Laser, Wolfgang	Engelke, David
Denamur, Erick	Drummond, Alexei	Engels, William
Deng, Hong-Wen	Drummond, Iain	Epstein, Doug
Deng, Xing Wang	Drummond-Barbosa, Daniela	Erdman, Scott
Denning, David	Dubnau, David	Escribano, Julio
Denver, Dee	Dudbridge, Frank	Eskin, Eleazar
Dermitzakis, Emmanouil	Duncan, Ian	Essex, Max
Dernburg, Abby	Duncan, Stephen	Esteller, Manel
DeSalle, Robert	Dunham, Ian	Estivill, Xavier
Desplan, Claude	Dunham, Maitreya	Estoup, Arnaud
Dessein, Alain	Duquesnoy, Rene	Etges, William
Detter, Chris	Duret, Laurent	Etzel, Carol
Deutsch, Samuel	Duronio, Robert	Eulgem, Thomas
Deutschbauer, Adam	Dutheil, Julien	Evan, Gerard
Devlin, Bernie	Dyer, Kelly	Evans, David
Devoto, Steve	Dyson, Nick	Evans, Ronald
Devriendt, Koen	Earnshaw, Bill	Evans, Thomas C.
Di Rienzo, Anna	Eastman, Deborah	Ewbank, Jonathan
Dickson, Barry	Eberhart, Charles	Ewens, Warren
Didelot, Xavier	Ebers, George	Excoffier, Laurent
Dieckmann, Carol	Eddy, Sean	Eyras, Eduardo
Dietrich, William	Edwards, Robert	Eyre-Walker, Adam
Dietz, Harry	Egly, Jean-Marc	Fago, Angela
Dill, David	Eickbush, Tom	Fain, Pamela
Dillin, Andy	Eide, David	Fairbrother, Will
Dina, Christian	Eilers, Martin	Fajas, Lluis
Ding, Jun	Eisen, Jonathan	Falush, Daniel
Dinman, Jonathan	Eisen, Michael	Fan, Hung
Disteche, Christine	Eisenbarth, George	Farese, Robert
Divers, Jasmin	Eisenberg, Eli	Farnham, Peggy
Dixon, Michael J	Ekker, Stephen	Farooqi, Sadaf
Dobrindt, Ulrich	Elde, Nels	Farr, Christine
Doebley, John	Elgar, Greg	Farrall, Martin
Doherty, Aidan	Elgin, Sarah	Fay, Justin
Dohlman, Henrik	Elledge, Steve	Fearnhead, Paul
Donaldson, Anne	Ellerby, Lisa	Feder, Martin
Donehower, Larry	Elliot, Rosemary	Feil, Edward
Donohue, Timothy	Ellis, Ronald	Feil, Robert
Dorit, Rob	El-Shanti, Hatem	Feinberg, Andrew
Douzery, Emmanuel	Emerson, Stephen	Fekete, Donna
Dowling, John	Emery, Patrick	Feldgarden, Michael
Draper, Bruce	Emeson, Ronald	Feldman, Marcus

Felix, Marie-Anne	Freedman, Jonathan	Gibson, Greg
Ferguson-Smith, Anne	Friedberg, Errol	Giguere, Vincent
Fernandez, Jose	Friedman, Robert	Gilad, Yoav
Ferreira, Manuel	Friedman, Thomas	Gilbert, Nicolas
Fersht, Alan	Friedrich, Markus	Gillespie, Peter
Feschotte, Cedric	Froguel, Philippe	Gilliland, Frank
Festenstein, Richard	Fruman, David	Gingeras, Tom
Ffrench-Constant, Richard	Fry, James	Glaser, Philippe
Fiering, Steven	Fugger, Lars	Glass, N. Louise
Filatov, Dmitry	Fukaki, Hidehiro	Glazer, Peter
Fingerlin, Tasha	Fuller, Margaret	Gloor, Greg
Fink, Gerald	Furano, Anthony	Glover, Thomas
Finkbeiner, Steven	Furutani-Seiki, Makoto	Glusman, Gustavo
Finkelstein, David	Gaiano, Nicholas	Göbel, Thomas
Finlayson, Scott	Galán, Jorge	Goddard, Michael
Finnegan, David	Galhardo, Rodrigo	Goeman, Jelle
Finnegan, E. Jean	Gall, Joseph	Goff, Stephen
Firtel, Rick	Gallant, Peter	Gojobori, Takashi
Fischer, Alain	Gallo, Jean-Marc	Golbeck, John
Fischer, Daniel	Galtier, Nicolas	Gold, Ralf
Fischer, Gilles	Gao, Fen-Biao	Golden, Andy
Fisher, Elizabeth	Gao, Guimin	Goldowitz, Dan
Fisher, Matthew	Garcia, Emilio	Golic, Kent
Fisher, Simon	Garcia, L. Rene	Gollin, Susanne
Fitzpatrick, Ben	Garcia-Blanco, Mariano	Goodell, Margaret
Fleischmann, Bernd	Garner, Chad	Goodman, Steve
Fletcher, Colin	Garrett, Roger	Goodrich, Justin
Flicek, Paul	Garrigan, Dan	Goodrich, Lisa
Flint, Jonathan	Gasch, Audrey	Goodwin, Stephen
Florea, Liliana	Gasser, Susan	Goossens, Alain
Florez, Jose	Gatz, Christiane	Gordenin, Dmitry
Foiani, Marco	Gaudet, Jeb	Gordo, Isabel
Foley, Edan	Gaut, Brandon	Gordon, Derek
Fondon, John	Geballe, Adam	Gorin, Michael
Foote, Simon	Gelb, Bruce	Göring, Harald
Ford, James	Gems, David	Gossler, Achim
Forsburg, Susan	Georges, Michel	Gotoh, Osamu
Francino, M. Pilar	German, Michael	Gottesman, Susan
François, Olivier	Gerstein, Mark	Gottlieb, Eyal
Franke, Lude	Gerton, Jennifer	Gottschling, Daniel
Frasch, Manfred	Geschwind, Dan	Gould, Kathleen
Fraser, Peter	Gharavi, Ali	Grabowski, Paula
Fraser-Liggett, Claire	Ghosh, Debashis	Graham, Anthony
Frayling, Tim	Giacca, Mauro	Grandori, Carla
Frazer, Kelly	Gibbs, Richard	Grange, Thierry

Grant, Sarah	Haiman, Christopher	Hebebrand, Johannes
Graur, Dan	Haines, Jonathan	Heber, Steffen
Graveley, Brenton	Hajnal, Alex	Heck, Margarete
Graves, Barbara	Hake, Sarah	Hedrick, Phil
Gray, Joe	Halder, Georg	Heidmann, Thierry
Grayhack, Elizabeth	Halfon, Marc	Heinzen, Robert
Grbic, Miodrag	Hall, Barry	Heisenberg, Carl-Philipp
Greally, John	Halliday, Karen	Heitman, Joseph
Green, Carla	Halligan, Daniel	Helgason, Agnar
Greene, Eric	Halpern, Marnie	Helin, Kristian
Greenstein, David	Hamblin, Martha	Hellmann, Ines
Grewal, Shiv	Hamiche, Ali	Hendershot, Linda
Grigorenko, Elena	Hamilton, Bruce	Henderson, Ian
Grigoriev, Igor	Hammer, Michael	Hendrich, Brian
Grishok, Alla	Hammerschmidt, Matthias	Hendrickson, Eric
Groden, Joanna	Hampe, Jochen	Hendrickson, Heather
Groisman, Alex	Han, Min	Henikoff, Steven
Gros, Philippe	Hanawalt, Philip	Hennig, Branwen
Grosovsky, Andrew	Hancock, John	Hennig, Lars
Grosveld, Frank	Handel, Mary Ann	Henry, Clarissa
Grozinger, Christina	Hanks, Steve K.	Herman, Bob
Gruber, Steve	Hannon, Gregory J.	Herman, Christophe
Gu, Xun	Hansen, Malene	Hermisson, Joachim
Gu, Zhenglong	Hardin, Jeff	Hernandez, Nouria
Gudjonsson, Johann	Hardison, Ross	Hernandez, Ryan
Guenet, Jean-Louis	Hardwick, Kevin	Herr, Winship
Guerin, Gerard	Hardy, John	Herrick, Glenn
Guerreiro, J. F.	Harfe, Brian	Heslop-Harrison, J. Pat
Guigo, Roderic	Harland, Richard	Hess, Wolfgang
Guillot, Gilles	Harris, Reuben	Hey, Jody
Gunzl, Arthur	Harris, Tony	Heyer, Evelyne
Gusella, James	Harrison, Douglas A.	Heyer, Wolf
Gut, Ivo	Harshman, Lawrence	Heyting, Christa
Guttman, David	Harushima, Yoshiaki	Hide, Winston
Gvozdev, Vladimir	Hassold, Terry	Hieter, Philip
Gyllensten, Ulf	Hatzigeorgiou, Artemis	Higgins, N. Pat
Haber, James	Hauser, Elizabeth	Higgs, Doug
Hacker, Joerg	Hauser, Loren	Hill, William
Hadany, Lilach	Haussler, David	Hillert, Jan
Hadly, Elizabeth	Hawley, R. Scott	Hillis, David
Hafler, David	Hay, Bruce	Hinds, David
Hagan, Iain	Hayashizaki, Yoshihide	Hinds, Philip
Hahn, Matthew	Hays, Thomas	Hingorani, Sunil
Hahn, Steve	Heard, Edith	Hinnebusch, Joseph
Hahn, William	Heath, Simon	Hirano, Michio

Hirotsune, Shinji	Hsieh, Wen-Ping	Jackson, George
Hirschhorn, Joel	Hsu, Ellen	Jackson, Ian
Hirsh, Jay	Huang, Hai	Jackson, Mike
Hitzemann, Robert	Huang, Jing	Jacobsen, Steven
Hlusko, Leslea	Hubner, Norbert	Jacobson, Allan
Ho, Simon	Hudson, Andrew	Jaenike, John
Hobbs, Charlotte	Hudson, Richard	Jain, Rakesh
Hobert, Oliver	Hudson, Thomas	James, Timothy
Hochwagen, Andreas	Huelsenbeck, John	Janne, Pasi
Hodgkin, Jonathan	Hughes, Anne	Jansen, Ritsert
Hoeijmakers, Jan	Hughes, Austin	Jantsch, Michael
Hoekstra, Hopi	Hughes, Cynthia	Jasper, Heinrich
Hoekstra, Rolf	Hughes, Jennifer	Jaspersen, Sue
Hofacker, Ivo	Hughes, Laura	Jeck, William
Hoffmann, Ary	Hughes, Stacie	Jeffreys, Alec
Hoffmann, Hans	Hughes, Timothy	Jenkins, Gareth
Hofreiter, Michael	Hungria, Mariangela	Jenkins, Nancy
Hogenesch, John	Hunt, Patricia	Jezek, Petr
Hoggart, Clive	Huq, Enamul	Jiang, Ning
Holger, Puchta	Hurles, Matthew	Jiang, Yun-Jin
Holland, Eric	Hurst, Laurence	Jiao, Yuling
Holley, Matthew	Huttner, Wieland	Jin, Peng
Hollingshead, Susan	Hyman, Anthony	Jinks-Robertson, Sue
Hollingsworth, Nancy	Ideker, Trey	Johannes, Frank
Holloway, Alisha	Ikeda, Akihiro	Johansen, Kristen
Holmes, Chris	Imai, Shin-ichiro	John, Hardy
Holmes, Edward	Ingham, Philip	Johnson, Jason
Holmes, Scott	Ingraham, Holly	Johnson, Ken
Holt, Rob	Innan, Hideki	Johnson, Norman
Holzbaur, Erika	Ira, Grzegorg	Johnson, Stephen
Hong, Kyonsoo	Iraqi, Fuad	Johnson, Thomas
Honig, Lawrence	Iritani, Brian	Johnson, Toby
Höög, Christer	Isberg, Ralph	Johnson, Tom
Hoover-Fong, Julie	Ischiropoulos, Harry	Johnston, Laura
Horie, Kyoji	Ishino, Fumitoshi	Johnstone, Karen
Horn, Matthias	Isles, Anthony	Jones, Corbin
Hornstein, Eran	Issa, Jean-Pierre	Jones, David
Horvath, Steve	Ivanova, Natalia	Jones, Gareth H.
Hosoya, Hiroshi	Ivics, Zoltan	Jones, Peter
Houart, Corinne	Iyer, Vishwanath	Jonkers, Jos
Howard, Michael	Izaurralde, Elisa	Jordan, I. King
Howie, Bryan	Izpisúa Belmonte, Juan Carlos	Jorgensen, Eric
Hoyt, M. Andrew	Jacks, Tyler	Jornsten, Rebecka
Hrabé de Angelis, Martin	Jackson, Andrew	Joseph, Sarah
Hsieh, Peggy	Jackson, F. Rob	Juenger, Thomas

Junakovic, Nikolaj	Kelly, Bill	Kocher, Tom
Justice, Monica	Kelsey, Gavin	Koga, Akihiko
Kaback, David	Kelsh, Robert	Kögel, Donat
Kaeberlein, Matt	Kendziorski, Christina	Kohn, Linda
Kaeppler, Shawn	Kenmochi, Naoya	Kolodkin, Alex L.
Kaessmann, Henrik	Kennedy, Brian	Kondo, Shinji
Kaitila, Ilkka	Kent, Jack	Kondo, Shu
Kakutani, Tetsuji	Kenyon, Cynthia	Kondoh, Hisato
Kalderon, Daniel	Kernan, Maurice	Kondrashov, Alex
Kallipolitis, Birgitte	Kerr, Kathleen	Kondrashov, Fyodor
Kamakaka, Rohinton	Ketting, René	Koob, Mike
Kamboh, M. Ilyas	Khaitovich, Philipp	Koonin, Eugene
Kammenga, Jan	Khochbin, Saadi	Kooperberg, Charles
Kamoun, Sophien	Kidson, Susan	Koornneef, Maarten
Kanaar, Roland	Kim, Joomyeong	Kopp, Artyom
Kanapin, Alexander	Kim, Junhyong	Korey, Christopher
Kang, Yibin	Kim, Kyoungmi	Kornblihtt, Alberto
Kapahi, Pankaj	Kim, Narry	Kornfeld, Kerry
Kapitonov, Vladimir	Kim, Seung	Korolev, Sergey
Kaplan, Joshua	Kim, Stuart	Korzh, Vladimir
Karch, François	Kim, Su Yeon	Koshland, Douglas
Kardia, Sharon	Kim, Tae Hoon	Kosiol, Carolin
Karl, David	Kim, Yuseob	Kowalczykowski, Stephen
Karlin, Sam	Kimelman, David	Kozak, Leslie
Karpen, Gary	Kimmel, Charles	Kraft, Peter
Karr, Tim	Kimmel, Marek	Krauthammer, Michael
Kassem, Moustapha	Kinzy, Terry	Krek, Wilhelm
Katsanis, Nicholas	Kirkpatrick, Mark	Krieg, Paul
Katsura, Isao	Kirkup, Benjamin	Krogan, Nevan
Kaufmann, William	Kittles, Rick	Krol, Alain
Kauppi, Liisa	Kivisild, Toomas	Kronstad, James
Kay, Steve	Kladde, Michael	Kruglyak, Leonid
Kazazian, Haig	Kleckner, Nancy	Kruglyak, Semyon
Keavney, Bernard	Klein, Franz	Kües, Ursel
Kee, Barbara	Klein, Hannah	Kui, Zhang
Keeney, Scott	Kliebenstein, Daniel	Kulathinal, Rob
Kehrer-Sawatzki, Hildegard	Knapik, Ela	Kulkarni, Rohit
Keightley, Peter	Knight, Joanne	Kültz, Dietmar
Keinan, Alon	Knight, Kendall	Kumar, Sudhir
Kel, Alexander	Knight, Robert	Kummerfeld, Sarah
Keller, Evan	Knight, Samantha	Kunisada, Takahiro
Keller, Mathew	Knipple, Doug	Kunkel, Gary
Kelley, Mathew	Knop, Michael	Kuroda, Mitzi
Kelley, Rick	Knust, Elisabeth	Kusche-Gullberg, Marion
Kellis, Manolis	Kobayashi, Ichizo	Kwiatkowski, Dominic

Kwok, Pui-Yan	Lee, Maxwell	Lipovich, Leonard
La Spada, Albert	Lee, Mike	Lipshitz, Howard
Labouesse, Michel	Lee, Sang Eun	Lisch, Damon
Ladurner, Andreas	Lee, Siu Sylvia	Lister, Jim
Lafontaine, Denis	Leeb, Tosso	Lithgow, Gordon
Lahn, Bruce	Lefkowitz, Elliot	Litman, Gary
Lahue, Robert	Lehmann, Alan	Little, Tom
Laird, Peter	Leibel, Rudolph	Liu, Dawei
Lake, Cathy	Leibovici, Michel	Liu, Edison
Lambert, J. David	Lemire, Mathieu	Liu, Jun
Lambris, John	Lemischka, Ihor	Liu, Nianjun
Lammer, Edward	Lemmon, Sandra	Livingston, Dennis
Land, Hartmut	Lemos, Bernardo	Llopart, Ana
Lane, David	Lenhard, Boris	Lloyd, Robert
Lane, E. Birgitte	Leonard, Jennifer	Logsdon, John
Lane, Mary Ellen	Leonetti, Jean-Paul	Lohmueller, Kirk
Lane, Robert	Leptin, Maria	Loidl, Josef
Langefeld, Carl	Lercher, Martin	Loisel, Dagan
Langley, Charles	Lesage, Pasqual	Londei, Paola
Lansdorp, Peter	Lesueur, Fabienne	Londono-Vallejo, J. Arturo
Lapidus, Alla	Lettice, Laura	Long, Anthony
Lareau, Liana	Letts, Verity	Long, Jeff
Largaespada, David	Levine, Michael	Long, Manyuan
Larhammer, Dan	Levy, Sasha	Longhese, Maria Pia
Lark, K. Gordon	Levy, Shawn	Longnecker, Daniel
Larsson, Jan	Lewandowski, Mark	Longo, Nicola
Larsson, Nils-Göran	Lewis, Suzanna	Longo, Valter
Larsson, Tobias	Li, Hongzhe	Lopez, Bernard
Larue, Lionel	Li, Mingyao	Lopez, Rubens
LaSalle, Janine	Liang, Liming	Loraine, Ann
Lascoux, Martin	Liblau, Roland	Lorincz, Matthew
Laskey, M. Ann	Licht, Jonathan	Louis, Christos
Lasko, Paul	Lichten, Michael	Louis, Ed
Lau, Gee	Lichter, Peter	Lovett, Michael
Laudet, Vincent	Lieb, Jason	Lovett, Susan
Laurie, Cathy	Liebman, Susan	Low, K Brooks
Lawrence, Jeanne	Lilly, Mary	Lowell, Brad
Lawrence, Jeffrey	Lin, Danyu	Lu, Bingwei
Lazzaro, Brian	Linardopoulou, Elena	Lucas, Robert
Leach, David	Lind, Penelope	Lucchesi, John
Leahy, Dan	Linial, Maxine	Lukaszewski, Adam
Leal, Suzanne	Link, Andrew	Lundblad, Vicki
Lee, Brendan	Link, Chris	Lundquist, Erik
Lee, Charles	Link, Daniel	Lunter, Gerton
Lee, Jeannie	Lipkin, Steven	Luscombe, Nicholas

Lusetti, Shelley	Maraganore, Demetrius	McKee, Bruce
Lusis, Aldons	Marahrens, York	McKim, Kim
Lustig, Art	Maraia, Richard	McMahon, Andy
Lydall, David	Marais, Gabriel	McMullen, Michael
Lyko, Frank	Marais, Richard	McPeek, Mary
Lyle, Stephen	Marchini, Jonathan	McQueen, Matthew
Lyons, Karen	Marcotte, Edward	McRae, Allan
Ma, Hong	Marine, Jean-Christophe	McVean, Gil
Maas, Richard	Marini, Joan	McVey, Mitch
Macaulay, Vincent	Marinus, Martin	Mead, Simon
MacDonald, Marcy	Marjoram, Paul	Meehan, Richard
MacGregor, Stuart	Marrack, Philippa	Meeley, Robert
Machesky, Laura	Marsh, Steve	Megee, Paul
Mackay, Ian	Martienssen, Robert	Megeney, Lynn
Mackay, Trudy	Martin, James	Meisler, Miriam
Mackinnon, Margaret	Martin, Mark	Meller, Victoria
MacPherson, David	Martin, Rennee	Meltzer, Paul
Madeo, Frank	Masel, Joanna	Mendell, Joshua
Madhani, Hiten	Maskell, Duncan	Mendelsohn, Cathy
Maga, Giovanni	Masson, Jean-Yves	Mergeay, Max
Mager, Dixie	Matic, Ivan	Merry, Brian
Maggert, Keith	Matise, Tara	Mersha, Tesfaye
Maggi, Adriana	Mattick, John	Meshorer, Eran
Magnuson, Terry	Mattox, William	Mette, Florian
Maher, Veronica	Matzke, Marjori	Metzenberg, Aida
Mahowald, Anthony	Maue, Robert	Metzger, Daniel
Maiden, Martin	Maurelli, Anthony	Metzstein, Mark
Maizels, Nancy	May, Georgiana	Meuwissen, Theo
Makalowski, Wojciech	Mayer, Bruce	Meyer, Barbara
Makarova, Kira	Mazel, Didier	Meyer, Christian
Makova, Kateryna	McAllister, Bryant	Meyer, Peter
Malik, Harmit	McArdle, Jack	Meyerhof, Wolgang
Malik, Punam	McCarrey, John	Meyers, Blake
Malkova, Anna	McCarroll, Steven	Meyn, M. Stephen
Mallet, Jim	McCarson, Kenneth	Michel, Bénédicte
Man, Orna	McCarthy, Jeanette	Middleton, Derek
Mander, Adrian	McCarthy, Mark	Mikkola, Marja
Manfredi, Jim	McCollum, Dannel	Milinski, Manfred
Mangelsdorf, David	McCusker, John	Millar, Sarah
Mangion, Jonathan	McDonald, John	Miller, Charles
Mango, Susan	McGhee, James	Miller, David
Mankin, Alexander	McGregor, Alistair	Miller, Marcia
Manley, Nancy	McGuffin, Liam	Miller, Michael
Mann, Matthias	McIntire, Steve	Miller, Richard
Mansfield, John	McIntyre, Lauren	Miller, Robert

Miller, Webb	Muglia, Louis	Neuberger, Michael
Miller, Wilson	Muller, Ferenc	Neumann, Carl
Mills, Alea	Muller, Fritz	Newton, Michael
Mills, Kevin	Muller, Jurg	Newton-Cheh, Christopher
Minami, Yasuhiro	Mullins, Mary	Nicholas, Frank
Minion, Chris	Munafo, Marcus	Nickerson, Deborah
Mirnics, Karoly	Mundlos, Stefan	Nicolae, Dan
Miska, Eric	Mundy, Nick	Nielsen, Dahlia
Misteli, Tom	Munroe, Patricia	Nielsen, Einar
Mitchell, Aaron	Murphy, Coleen	Nielsen, Henrik
Mitchell-Olds, Thomas	Murphy, Keith	Nielsen, Rasmus
Mlodzik, Marek	Murphy, Susan	Niki, Hironori
Mockler, Todd	Murphy, William	Nikolaidis, Nikolas
Moens, Peter	Murray, Jeffrey	Nilsen, Timothy
Moghal, Nadeem	Murray, Johanne	Nishikura, Kazuko
Mogil, Jeffrey	Murray, Noreen	Nishimura, Taisuke
Mohlke, Karen	Musani, Solomon	Nishina, Patsy
Mombaerts, Peter	Muscatelli, Françoise	Nislow, Corey
Montgomery, Stephen	Myers, Garry	Niswander, Lee
Montpetit, Alexandre	Myers, Richard	Nitabach, Michael
Moore, Tom	Myers, Simon	Nizetic, Dean
Mootha, Vamsi	Myung, Kyungjae	Nobrega, Marcelo
Moran, John	Nachman, Michael	Noor, Mohamed
Moran, Mary Ann	Nadeau, Jeanette	Nordborg, Magnus
Morgan, Bruce	Nagata, Kyosuke	Norga, Koenraad
Mori, Kazutoshi	Naggert, Jurgen	Norris, Steven
Mori, Yasuo	Nagy, Andras	North, Kari
Morrell, Peter	Nakahigashi, Kenji	Nothnagel, Michael
Morris, Andrew	Nasrallah, Mikhail	Novembre, John
Morris, Kevin	Nath, Swapan	Nugent, Constance
Morrison, Ciaran	Nathanson, Katherine	Nui, Tianhua
Morrissette, Naomi	Navarro, Arcadi	Nunez, Gabriel
Morrow, Bernice	Neafsey, Daniel	Nunnari, Jodi
Morton, Newton	Neale, Benjamin	Nuzhdin, Sergey
Moses, Alan	Neale, Matthew	Nyholt, Dale
Moses, Kevin	Need, Anna	Nyström, Thomas
Mott, Richard	Neel, Benjamin	Obara, Tomoko
Mountain, Joanna	Neil, Jim	Obbard, Darren
Muchowski, Paul	Nelson, David	Ober, Carole
Muegge, Kathrin	Nelson, John	O'Brien, Stephen
Mueller, Arno	Nelson, Peter	Ochman, Howard
Mueller, Juerg	Nelson, Stanley	O'Donnell, Chris
Mueller, Lukas	Nelson, Timothy	O'Farrell, Patrick
Mueller-Myhsok, Bertram	Neri, Christian	Ogas, Joe
Muenke, Max	Netea, Mihai	O'Grady, Patrick

Ohkura, Hiro	Pallen, Mark	Pereira, Vini
Ohler, Uwe	Palmer, Abraham	Pereira-Leal, Jose
Ohlsson, Rolf	Palmer, Jeff	Perrimon, Norbert
O'hUigin, Colm	Palmiter, Richard	Peterfy, Miklos
Okada, Norihiro	Palsboll, Per	Peters, Antoine
Okamura, Hitoshi	Palzkill, Timothy	Peters, Jan-Michael
Olivas, Wendy	Panda, Satchidananda	Peters, Jo
Oliver, Brian	Pandolfi, Pier Paolo	Peters, Luanne
Oliver, Steve	Pankratz, Michael	Petersen-Mahrt, Svend
Olsen, Kenneth	Panning, Barbara	Peterson, Craig
Olshen, Adam	Panthier, Jean-Jacques	Peterson, Thomas
Olshen, Richard	Papaioannou, Virginia	Petkov, Petko
Olson, James	Papatsenko, Dmitri	Pevzner, Pavel
Olson, Maynard	Papp, Balázs	Pfeifer, Gerd
O'Neill, Scott	Parada, Luis	Pfister, K. Kevin
Onel, Susanne-Filiz	Parham, Peter	Pfisterer, Andrea
Op den Camp, Huub	Paria, Bibhash	Piano, Fabio
Oppenheimer, Stephen	Parichy, David	Piatti, Simonetta
O'Rahilly, Steve	Parker, Roy	Pieper, Russell
Orlando, Valerio	Parkhill, Julian	Pillai, Sreekumar
Oro, Tony	Paro, Renato	Pillus, Lorraine
Orr, Harry	Parsch, John	Pilpel, Yitzhak
Orr-Weaver, Terry	Partridge, Janet	Pintard, Lionel
Orstavik, Karen Helene	Partridge, Linda	Pires, Chris
Ory, Dan	Parvin, Jeffrey	Pittelkow, Mark
Osborne, Cameron	Pasquinelli, Amy	Plagnol, Vincent
Osborne, Lucy	Passtoors, Willemijn	Planet, Paul
Oshima, Yasumi	Paterson, Andrew	Plant, Nick
Osley, Mary-Ann	Patterson, Nick	Plass, Christoph
Oster, Henrik	Paulson, Henry	Plasterk, Ronald
Ostrander, Elaine	Paulson, Robert	Pletcher, Scott
Otha, Kunihiro	Pavan, Bill	Plotkin, Joshua
Ott, Jurg	Payseur, Bret	Pluschke, Gerd
Ouchi, Toru	Pazour, Gregory	Poinar, Hendrik
Ovcharenko, Ivan	Pearl, Laurence	Pollack, Joshua
Owen, Art	Pearson, Christopher	Pollak, Martin
Owen-Hughes, Tom	Peaston, Anne	Pollard, Katherine
Pääbo, Svante	Pe'er, Dana	Polyak, Kornelia
Pachter, Lior	Pe'er, Itsik	Pombo, Ana
Padilla, Miguel	Peichel, Catherine	Ponting, Chris
Pagano, Michele	Peifer, Mark	Pool, John
Page, David	Pellegrini, Luca	Posakony, Jim
Page, Scott	Pellman, David	Poss, Ken
Pak, Bill	Peltz, Gary	Postlewait, John
Pál, Csaba	Penny, David	Potier, Marie-Claude

Potts, Wayne	Rando, Oliver	Riley, Monica
Poulter, Russell	Ranganathan, Shoba	Rine, Jasper
Pourquié, Olivier	Ranish, Jeffrey	Ringrose, Leonie
Pradhan, Sriharsa	Ranum, Laura	Ringwald, Martin
Prat, Salome	Ranz, Jose	Rinn, John
Predki, Paul	Rast, Jonathan	Rioux, John
Prentice, Micheal	Raymond, Pamela	Ripatti, Samuli
Price, Alkes	Read, Tim	Risques, Rosana
Price, Arlen	Rebbeck, Tim	Robert, Francois
Price, Carolyn	Redd, Alan	Robey, Ellen
Price, Jeffrey	Reddel, Roger	Robins, Harlan
Price, Trevor	Redden, David	Robinson, Nicholas
Priess, James	Redfield, Rosemary	Robinson, Wendy
Prince, Victoria	Reed, Floyd	Rocchi, Mariano
Pringle, John	Reed, Laura	Rocha, Eduardo
Pritchard, Jonathan	Reenan, Robert	Rocke, David
Pritham, Ellen	Regev, Aviv	Rockman, Matthew
Promislow, Daniel	Reich, David	Rockmill, Beth
Proud, Christopher	Reik, Wolf	Roder, John
Provine, Will	Reilly, Cavan	Roeder, G. Shirleen
Przedborski, Serge	Reinke, Valerie	Roeder, Kathryn
Przeworski, Molly	Reitman, Marc	Rogaev, Evgeny
Ptak, Susan	Relman, David	Rogers, Alan
Puca, Annibale Alessandro	Remington, David	Rohde, Klaus
Purcell, Shaun	Ren, Bing	Rohwer, Forest
Puri, Pie Lorenzo	Renfree, Marilyn	Rokas, Antonis
Purugganan, Michael	Renkawitz, Rainer	Romesberg, Floyd
Qin, Zhaohui	Renkawitz-Pohl, Renate	Ron, David
Quackenbush, John	Renne, Rolf	Rong, Yikang
Queitsch, Christine	Reppert, Steve	Roopenian, Derry
Queller, David	Rethwilm, Axel	Rosen, Clifford
Rabinowitz, Daniel	Reusch, Thorsten	Rosen, Jeffrey
Rafalski, Antoni	Reuter, Gunter	Rosenberg, Noah
Raghuraman, M.	Reverter-Gomez, Antonio	Rosenberg, Susan
Ragoussis, Jiannis	Reysenbach, Anna-Louise	Rosmarin, Alan
Rahman, Nazneen	Rhind, Nick	Ross, Mark
Raible, David	Richard, Gabriele	Ross, Susan
Raina, Ramesh	Richards, Eric	Rossant, Janet
Rakyan, Vardhman	Richardson, Guy	Roth, Frederick P.
Ramachandran, Sohini	Ricquier, Daniel	Roth, John
Ramakrishnan, Lalita	Riddiford, Lynn	Rothman, Joel
Ramaswami, Mani	Riddle, Brett	Rothstein, Rodney
Ramsden, Dale	Riess, Olaf	Rotig, Agnes
Rana, Brinda	Riethman, Harold	Rotimi, Charles
Rand, David	Riha, Karel	Rougeulle, Claire

Roussel, Martine	Sawyer, Stanley	Seielstad, Mark
Rowen, Lee	Saxena, Richa	Sekelsky, Jeff
Roy, Peter	Scanlan, David	Sekiguchi, JoAnn
Roy, Scott	Schadt, Eric	Selker, Eric
Rozen, Steve	Schaeffer, Stephen	Sémon, Marie
Rubinsztein, David	Schaffner, Steve	Sen, Ganes
Rudd, Katie	Schär, Primo	Sen, Saunak
Rudd, Kenneth	Schartl, Manfred	Sengupta, Piali
Ruden, Douglas	Schauwecker, Elyse	Serre, David
Rugarli, Elena	Schedl, Paul	Serreze, David
Ruley, H. Earl	Schedl, Tim	Service, Susan
Rulifson, Eric	Scheet, Paul	Servin, Bertrand
Runge, Kurt	Schejter, Eyal	Seto, Edward
Russell, Paul	Scherer, Stephen	Settleman, Jeffrey
Russell, Scott	Scheres, Ben	Sever, Sanja
Russell, Steve	Schibler, Ueli	Seydoux, Geraldine
Ruvkun, Gary	Schier, Alexander	Shackleton, Laura
Ryder, Oliver	Schierup, Mikkel	Shah, Parantu
Sabatti, Chiara	Schimenti, John	Shah, Sohrab
Sabeti, Pardis	Schisa, Jennifer	Shaham, Shai
Sadelain, Michel	Schlenke, Todd	Shakes, Diane
Sado, Takashi	Schloegelhofer, Peter	Shannon, William
Saetre, Peter	Schloetterer, Christian	Shapiro, Beth
Saez, Enrique	Schmid, Roland	Shapiro, James
Saga, Yumiko	Schmidt, Silke	Sharbel, Timothy
Sage, Julien	Schmidt-Ott, Urs	Sharma, Govind
Sainudiin, Raazesh	Schmucker, Dietmar	Sharp, Andy
Salome, Patrice	Schmutz, Jeremy	Shaw, Marie-Anne
Salomon, David	Schmutz, Sheila	Shay, Jerry W.
Salz, Helen	Schneider, David	Sheffield, Val
Salzberg, Steven	Schneitz, Kay	Shen, Kang
Sandhu, Manjinder	Schoenherr, Chris	Shepard, Allan
Sandmeyer, Suzanne	Schork, Nicholas	Sherlock, Gavin
Sang, Tao	Schreiber, Stefan	Shete, Sanjay
Sanna, Serena	Schübeler, Dirk	Shifman, Sagiv
Santos, Juan	Schueler, Mary	Shilatifard, Ali
Sapienza, Carmen	Schwab, Sibylle	Shimamoto, Ko
Sasaki, Hiroyuki	Schwob, Etienne	Shimeld, Sebastian
Sauer, Frank	Scott, Laura	Shimizu, Hiroshi
Saumweber, Harald	Scully, Ralph	Shinohara, Akira
Saunders, Nigel	Seehausen, Ole	Shiu, Shin-Han
Savage, Sharon	Segal, Eran	Shmulevich, Ilya
Sawa, Hitoshi	Segal, Marisa	Shoulders, Carol
Sawcer, Stephen	Segil, Neil	Shriner, Daniel
Sawyer, Sara	Sehgal, Amita	Shriver, Mark

Shuldiner, Alan	Sonenshein, Gail	Steward, Ruth
Sible, Jill	Sorek, Rotem	Stillman, Bruce
Sibley, L. David	Soriano, Philippe	Stock, David
Sidow, Arend	Southan, Christopher	Stoll, Monika
Siepel, Adam	Spangrude, Gerald	Stoltzfus, Arlin
Siggia, Eric	Spector, Tim	Stone, Anne
Sikela, James	Speer, Marcy	Stone, David
Silver, Simon	Spellman, Paul	Stoneking, Mark
Silverman, Gary	Spencer, Chris	Storb, Ursula
Simon, Conway	Spencer, Christine	Storey, John
Simon, Itamar	Spencer, Hamish	Stormo, Gary
Simpson, Julie	Spradling, Allan	Storz, Gisela
Sinclair, David	Spratt, Brian	Storz, Jay
Singh, Keshav	Springer, Mark	Stoye, Jonathan
Singh, Nadia	Springer, Nathan	Strähle, Uwe
Singh, Prim	St. Laurent III, Georges	Straight, Aaron
Singh, Rama	St.Clair, Dina	Stram, Daniel
Singleton, Andrew	Stacey, Gary	Stranger, Barbara
Siomi, Haruhiko	Stadler, Peter	Strehler, Emanuel
Siracusa, Linda	Staeheli, Peter	Strippoli, Pierluigi
Sjögren, Camilla	Stagljar, Igor	Strome, Susan
Skok, Jane	Stainier, Didier	Strub, Katharina
Skoultchi, Arthur	Stam, Maike	Struhl, Kevin
Slack, Frank	Stancheva, Irina	Stuart, Josh
Slack, Ruth	Stanier, Didier	Stuart, Philip
Slatkin, Monty	Stankiewicz, Pawel	Stubbs, Lisa
Slonim, Donna	Stankovich, Jim	Stunnenberg, Hendrik
Sluder, Ann	Stanley, Charles	Stupp, Roger
Smith, Andrew	Stanyon, Roscoe	Sturm, Richard
Smith, Des	Stasiak, Andrzej	Su, Tin Tin
Smith, Harold	States, David	Südhof, Thomas
Smith, Jeff	Stathopoulos, Angelike	Sue, Jaspersen
Smith, Susan	Statnikov, Alexander	Sugano, Sumio
Smith, Tanya	Stauber, Martin	Sugino, Akio
Smits, Guillaume	Stavnezer, Janet	Sullivan, Beth
Snyder, Larry	Steel, Mike	Sullivan, Chris
Sokolowski, Marla	Stefan, Norbert	Sullivan, Patrick
Söll, Dieter	Steimle, Viktor	Sulston, John
Solnica-Krezel, Liliana	Steinmetz, Lars	Sumazin, Pavel
Solter, Davor	Steitz, Joan	Summer, Elizabeth
Somero, George	Stemple, Derek	Summers, David
Somerville, Shauna	Stephan, Wolfgang	Sun, Tai-ping
Sommer, Ralf	Stephens, Matthew	Sun, Zhaoxia
Sonenberg, Nahum	Stern, David	Sundaram, Meera
Sonenshein, Abraham	Sternberg, Paul	Sundaresan, Venkatesan

Sunkel, Claudio	Thanaraj, Alphonse	Tyler-Smith, Chris
Sunyaev, Shamil	Thesleff, Irma	Tyson, John
Susan, Strome	Theurkauf, William	Udall, Joshua
Sutcliffe, James	Thomas, Stephen	Ueda, Hiroki R.
Sutter, Nathan	Thompson, Leslie	Urban, Nicole
Sutton, Mark	Thorne, Jeffrey L.	Valcarcel, Juan
Suzuki, Yoshiyuki	Thorne, Natalie	Valdar, William
Swanson, Maurice	Thornton, Kevin	Van der Bliek, Alex
Swanson, Willie	Threadgill, David	Van der Ende, Arie
Swaroop, Anand	Thummel, Carl	Van Doren, Mark
Sweasy, Joann	Thut, Cathy	Van Driel, Roel
Swedberg, Gote	Tillier, Elisabeth	Van Gent, Dik
Sweetlove, Lee	Tishkoff, Sarah	Van Gilst, Marc
Symington, Lorraine	Tissenbaum, Heidi	Van Houten, Bennett
Szyf, Moshe	Tiwari, Hemant	Van Lohuizen, Maarten
Tabin, Cliff	Tlsty, Thea	Van Melderen, Laurence
Taddei, Angela	Toczyski, David	Van Nimwegen, Erik
Takahashi, Joseph	Todd, John	Van Passel, Mark
Takahata, Naoyuki	Toledano, Michel	Van Steensel, Bas
Takeda, Eiji	Tomari, Yukihide	Vance, Vicki
Takeda, Junji	Tomkiel, John	Vanhaesebroeck, Bart
Takizawa, Peter	Toomajian, Christopher	Varga-Weisz, Patrick
Takumi, Toru	Tora, Laszlo	Varshavsky, Alexander
Talbot, William	Tower, John	Vasioukhin, Valeri
Tamkun, John	Townsend, Jeffrey	Vaughan, Duncan
Tan, Man-Wah	Tracey, Daniel	Vaughan, Laura
Tanay, Amos	Tran, Robert	Vaury, Chantal
Tang, Chao	Trask, Barbara	Vazquez, Franck
Tang, Hua	Traynor, Bryan	Veltman, Joris
Tang, Shanwu	Tremethick, David	Venkatesan, Sunaresan
Tanurdzic, Milos	Trifunovic, Aleksandra	Verkman, Alan
Tao, Qian	Triplett, Eric	Vermeire, Severine
Tapscott, Stephen	Tristem, Mike	Vermeulen, Wim
Tarakhovsky, Alexander	Trowsdale, John	Verrijzer, Peter
Tarazona-Santos, Eduardo	Troyanskaya, Olga	Vershon, Andrew
Tarsounas, Madalena	Trumpp, Andreas	Versteeg, Rogier
Tatar, Marc	Tsaih, Sharon	Verweij, Cor
Tavazoie, Saeed	Tsiantis, Miltos	Vetter, Monica
Taylor, Barbara	Tsukiyama, Toshio	Vicente-Carbajosa, Jesus
Taylor, Jennifer	Tunnacliffe, Alan	Vidal, Marc
Taylor, Steve	Turelli, Michael	Vidal-Puig, Antonio
Te Meerman, Gerard	Turnbull, Doug	Vieira, Cristina
Tempel, Bruce	Turner, Bryan	Vijg, Jan
Tenaillon, Olivier	Turner, James	Villeneuve, Anne
Tettelin, Hervé	Turner, Thomas	Vinogradov, Alexander

Vinson, Charles	Wasserman, Wyeth	Willer, Cristen
Visscher, Peter	Waterland, Robert	Williams, Julie
Vogel, Joerg	Watkins, Paul	Williams, Robert
Vogel, Joseph	Wayne, Robert	Williams, Rohan
Vogel, Ulrich	Weale, Michael	Williamson, Scott
Vogt, Peter	Weber, Michel	Willis-Owen, Saffron
Vogt, Tom	Weigel, Detlef	Wilson, Daniel
Volff, Jean-Nicolas	Weil, Cliff	Wilson, John
Vollrath, Doug	Weinberg, Eric	Wilson, Susan
Von Haeseler, Arndt	Weiner, Alan	Wilson, Tom
Voytas, Dan	Weinreich, Daniel	Winoto, Astar
Vundavalli, Murty	Weinstein, Brant	Wittbrodt, Joachim
Vyas, Paresh	Weir, Bruce	Wittkopp, Patricia
Wada, Hiroshi	Weiss, Eric	Wiuf, Carsten
Wade, Claire	Weiss, Ken	Wolfe, Kenneth
Wagner, Daniel	Weissman, Sherman	Wolfinger, Russell
Wagner, Doris	Welch, John	Wolfner, Mariana
Wagner, Günter	Welch, Steve	Wolkow, Catherine
Wakefield, Jonathan	Weller, Jennifer	Wong, Lee
Wakefield, Matthew	Weller, Roy	Wong, Wendy
Wakeley, John	Wellinger, Raymund	Woodard, Craig
Walbot, Virginia	Wells, Christine	Wootton, John C.
Walczak, Claire	Wells, Dan	Workman, Jerry
Waldman, Alan	Wendel, Jonathan	Worthington, Jane
Waldor, Matt	Weng, Nan-ping	Wray, Gregory
Walhout, Marian	Weng, Zhiping	Wray, Susan
Walker, Graeme	Werner, Andreas	Wren, Brendan
Walker, Graham	Wessler, Susan	Wright, Stephen
Walker, John	Wharton, Robin	Wu, Chung-I
Wall, Jeffrey	Wheeler, Diana	Wu, Doris
Wallenfang, Matt	White, Kenneth	Wu, Hong
Walsh, Bruce	White, Kevin	Wu, Rongling
Walter, Johannes	White, Rob	Wu, Tongtong
Walter, Jörn	Whitehead, Andrew	Wu, Xifeng
Wang, Haiyang	Whitelaw, Emma	Wuchty, Stefan
Wang, Jeremy	Whiteway, Malcolm	Wurst, Wolfgang
Wang, Jue	Whitfield, Michael	Wutz, Anton
Wang, Yufeng	Wieringa, Be	Wyckoff, Gerald
Wappner, Pablo	Wiest, David	Wynshaw-Boris, Anthony
Warburton, Dorothy	Wijmenga, Cisca	Wyrick, John
Warburton, Peter	Wilding, Jenny	Wysocka, Joanna
Warchol, Mark	Wilke, Claus	Xie, Gary
Ward, Robert	Wilkie, Andrew	Xie, Jun
Warden, Craig	Wilkins, Jon	Xie, Ting
Wassenegger, Michael	Willard, Huntington	Xu, Shizhong

Xu, Shunbin	Youngson, Neil	Zhang, Michael
Yaffe, Michael	Yu, C. Yung	Zhang, Xiaoyu
Yagi, Takao	Yu, Hongtao	Zhang, Ying
Yamagata, Kazuya	Yu, Jianming	Zhang, Zhaolei
Yamamoto, Daisuke	Yu, Xiao-Fang	Zhang, Zhuohua
Yamamoto, Keith	Yuhki, Naoya	Zhao, Hongyu
Yamashita, Akio	Yvert, Gael	Zhen, Mei
Yandell, Brian	Zamore, Phillip	Zheng, Tian
Yang, Hsiao-Pei	Zamoyska, Rose	Zhou, Bing
Yang, William	Zamponi, Gerald	Zhu, Heng
Yang, Ziheng	Zarkower, David	Zhu, Jian-Kang
Yankner, Bruce	Zavolan, Mihaela	Zhu, Xiaofeng
Yao, Meng-Chao	Zaykin, Dmitri	Zhuang, Yuan
Yasbin, Ronald	Zdobnov, Evgeny	Zhulin, Igor
Yelon, Deborah	Zeggini, Ele	Ziady, Assem
Yeo, Gene	Zeitlin, Scott	Zickler, Denise
Yi, Nengjun	Zeng, An-Ping	Ziegler, Andreas
Yoder, Bradley	Zeng, Zhao-Bang	Ziegler, Steven
Yonenaga-Yassuda, Yatiyo	Zetka, Monique	Zilberman, Daniel
Yoo, Yun Joo	Zeviani, Massimo	Zoellner, Sebastian
Yother, Janet	Zeyl, Clifford	Zon, Leonard
Youle, Richard	Zhang, Heping	Zuffardi, Orsetta
Young, Janet	Zhang, Jianzhi	Zuker, Charles
Young, Michael	Zhang, Kui	
